# Congenital Zika Virus Syndrome: Microcephaly and Orofacial Anomalies

**DOI:** 10.3390/life14010055

**Published:** 2023-12-28

**Authors:** Gaetano Scotto, Salvatore Massa, Francesca Spirito, Vincenzina Fazio

**Affiliations:** 1 Infectious Diseases Unit, University Hospital “OORR” Foggia, 71122 Foggia, Italy; 2 Department of Agriculture, Food, Natural Resource and Engineering, University of Foggia, 71122 Foggia, Italy; salvatore.massa@unifg.it; 3 Department of Clinical and Experimental Medicine, University of Foggia, 71122 Foggia, Italy; francesca.spirito@unifg.it; 4 Clinical Chemistry Laboratory, Virology Unit, University Hospital “OORR” Foggia, 71122 Foggia, Italy; enzafazio@gmail.com

**Keywords:** Zika virus, Aedes, pregnancy, CZS, microcephaly, orofacial anomalies

## Abstract

The progressive reappearance of Zika virus (ZIKV) infections since October 2013 and its circulation in >70 countries and territories (from French Polynesia to Brazil and other countries in the Americas, with sporadic spread in Europe and the East) has long been reported as a global public health emergency. ZIKV is a virus transmitted by arthropods (arboviruses), mainly by Aedes mosquitoes. ZIKV can also be transmitted to humans through mechanisms other than vector infection such as sexual intercourse, blood transfusions, and mother-to-child transmission. The latter mode of transmission can give rise to a severe clinical form called congenital Zika syndrome (CZS), which can result in spontaneous abortion or serious pathological alterations in the fetus such as microcephaly or neurological and orofacial anomalies. In this study, beside a succinct overview of the etiological, microbiological, and epidemiological aspects and modes of transmission of Zika virus infections, we have focused our attention on the pathogenetic and histopathological aspects in pregnancy and the pathogenetic and molecular mechanisms that can determine microcephaly, and consequently the clinical alterations, typical of the fetus and newborns, in a subject affected by CZS.

## 1. Introduction

In the last twenty years, we have increasingly witnessed the appearance/reappearance of previously unknown or known viral populations, often in epidemic form, which are pathogenic for humans. They are largely made up of the Arborvirus group (arthropod-borne viruses), viruses that require a specific vector that can interact with the vertebrate host. Various viral families belong to this group: Togaviridae, Flaviviridae, and Bunyaviridae are the main ones that cause human endemic/epidemic diseases [1]. This epidemic increase in Arborvirus infection is linked to various factors: climatic–environmental changes, the geographical spread of vectors, uncontrolled urbanization, global travel, and the receptivity of populations. Infections from some of these viruses, such as Dengue, Chikungunya, Zika, and West Nile Virus (WNV), are increasingly frequent globally and are now appearing in the Western Hemisphere. Indigenous manifestations of Dengue, Chikungunya epidemics, and the endemic circulation of WNV have been observed even recently in southern Europe, as well as WNV outbreaks in the Netherlands and Germany [2]. Among the Flaviviruses, the Zika virus (ZIKV) is assuming increasing importance due to the clinical implications it can cause. Already known in the first half of the last century due to sporadic cases in the African and Asian continents, the progressive epidemic expansion of the infection since 2013 in the Pacific islands, and in subsequent years in Brazil and other American countries, with infrequent occurrences in Europe, has long been reported as a possible global public health emergency [3]. ZIKV infection can often be completely asymptomatic or present clinically in a moderate form (short-term fever, headache, arthromyalgia [4], maculopapular rash, and/or non-purulent conjunctivitis). However, it can also cause serious clinical manifestations such as neurological pathologies (Guillain-Barrè syndrome), possible sequelae affecting the ocular system, and, above all, congenital fetal microcephaly. The latter is linked to vertical transmission during pregnancy, known as congenital Zika virus syndrome (CZS) [5]. CZS presents as a set of congenital defects including reduced brain size, brain anomalies, ocular anomalies, congenital contractures, intrauterine growth restriction, convulsions, pyramidal or extrapyramidal anomalies, delayed neurological development [6], and, in most cases, malformations and orofacial pathologies. In this study, beside a succinct overview of the etiological, microbiological, and epidemiological aspects and means of transmission of Zika virus infection, we have focused our attention on the pathogenetic and histopathological aspects in pregnancy and the pathogenetic and molecular mechanisms that can determine microcephaly, and consequently the clinical alterations, typical of the fetus and newborns, in a subject affected by CZS.

## 2. Zika Virus: Structure and Pathogenic Insights

The Zika virus is a Flavivirus, recently (April 2023) classified as belonging to the Orthoflavivirus genus Zikaense [7]. Zika is antigenically and structurally similar to the viruses that cause Dengue, Yellow fever, Japanese encephalitis, and WNV disease [8]. One of its characteristics, which differentiates it from other Flaviviruses, is its close phylogenetic relationship with the Spondweni virus. Both Zika and Spondweni form a real clade. ZIKV is classified as risk group 2 in Biosafety in Microbiological and Biomedical Laboratories [9].

**Figure 1:**
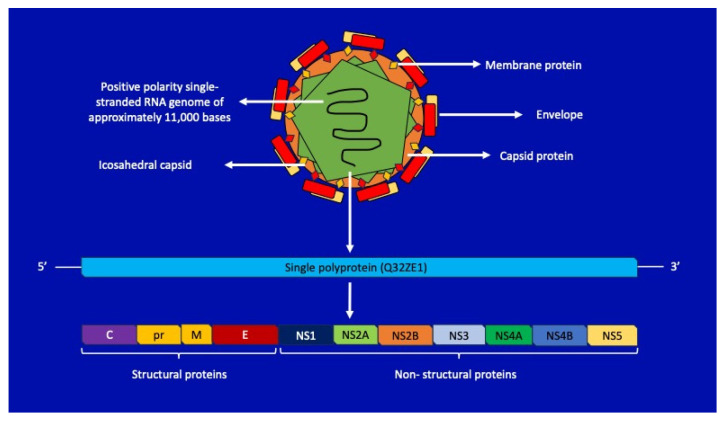
Zika virus genomic and capsid structure and encoded genes.

ZIKV has a spherical shape, with a diameter of no more than 40 nm, with a positive-polarity single-stranded RNA genome of approximately 11,000 bases enclosed in an icosahedral capsid. The genome is flanked by two untranslated regions, 5′ and 3′ UTR, which cooperate in viral replication [10]. The RNA is translated into a single polyprotein (Q32ZE1 genome polyprotein, 3423 amino acids in length) encoding seven non-structural proteins as well as three structural proteins [8]. The non-structural proteins, crucial for viral replication, include NS1 (considered the primary regulator), NS2A, NS2B, NS3, NS4A, NS4B, and NS5. Notably, NS5 functions as an RNA-dependent RNA polymerase, playing a key role in the virus’s replicative processes (Figure 1) [11]. Structural proteins (C, prM, and E) make up the physical structure of the virus, while non-structural proteins help replicate the genetic material, process the polyprotein, and control the interaction of the virus with the host. Among the structural proteins, the virus is encapsulated by the envelope glycoprotein; this protein initiates endocytosis by binding to the endosomal membrane of the host cell [12]. It is not yet completely clear how ZIKV enters and infects cells, but it is hypothesized that the first cells to be infected are the dendritic ones near the site of the infectious bite, with rapid subsequent spread to the lymph nodes and, afterwards, to the circulatory system. To replicate inside cells, ZIKV must overcome the barrier of receptor signals induced by type 1 IFN [13]; this occurs largely through the degradation of STAT2 (Signal Transducer and Activator of Transcription) signal molecules, the protein messenger cascade activated by the IFN receptor [14]. Once the cell is infected, the virus restructures the endoplasmic reticulum, resulting in the formation of large vacuoles and the death of the cell. One study highlighted that after six hours of ZIKV infection, vacuolization and the expansion of the mitochondria of infected cells begin [15]. This enlargement becomes so severe that it causes cell death, also known as paraptosis, and is strictly dependent on gene expression. Viruses cannot replicate until they infect and “reprogram” host cells. The epidermis and dermis of the host skin contain immature fibroblasts, keratinocytes, and dendritic cells, where ZIKV replication occurs [15].

## 3. Epidemiological Aspects

ZIKV was first isolated in Uganda in 1947 from Zika forest monkeys, after which it was named. In the 1950s, only sporadic human infections were reported, exclusively within a limited equatorial band from Africa to Asia. The first recognized case of infection in humans dates back to 1968 in Nigeria, and from 1968 to 2007, other human cases were recorded exclusively in Central Africa and Southeast Asia [16]. From 2007 to 2016, the virus spread eastwards across the Pacific Ocean to the American continent (mainly Brazil), causing various epidemic episodes. The first epidemic was reported in 2007 on the island of Yap (Micronesia), where 185 suspected cases were identified. Between 2013 and 2014, further epidemics occurred in several areas of the Pacific: French Polynesia, Easter Island, the Cook Islands, and New Caledonia. In the first four months of 2015, approximately 7000 suspected cases were reported in Brazil; the circulation of ZIKV in this country was confirmed in 2016; at the same time, there was a strong increase in suspected cases of neonatal microcephaly [17]. The increased spread of ZIKV infection and the possible association between the infection during pregnancy and the onset of microcephaly in newborns, as well as the correlation between the infection and other neurological disorders, pushed the WHO to declare in February 2016 an “international public health emergency” [18]. There are two different viral strains of ZIKV: African and Asian. The viral strain present in the Americas is 89% identical to African genotypes, but according to phylogenetic studies, it is more closely related to the Asian strain that circulated in French Polynesia during the 2013/14 pandemic [19]. After the 2015/16 epidemic, ZIKV transmission remained at lower levels. The WHO reports that as of July 2019, 87 countries and territories distributed across four of the six WHO regions have reported cases of autochthonous transmission from ZIKV. Additionally, in 61 countries and territories, the presence of competent vectors (Aedes aegypti) has been ascertained despite the absence of documented cases of disease. At the moment, there are no autochthonous outbreaks of vector-transmitted ZIKV in Europe, but the health authorities of several countries (Denmark, Finland, Germany, Holland, Portugal, the UK, Spain, and Sweden) have reported the presence of cases of infection in travelers returning from endemic areas [20]. Furthermore, sporadic cases of CZS have been described in subjects born in Europe to mothers who contracted the infection in endemic areas during the first months of pregnancy. Furthermore, a recent meta-analysis assessing transfusion-transmitted Zika virus risk in blood banks, performed on 528,947 blood samples, revealed an overall pooled prevalence of ZIKV infection in blood donations at 1.02% [21]. As regards Italy, data indicate that in the period between January 2020 and July 2023, there were five cases of ZIKV infection associated with travel abroad and one case of non-native CZS [20].

## 4. Transmission Mode

The ZIKV disease is typically a vector disease, which is mainly transmitted through mosquitoes of the Aedes genus, such as “*Aedes aegypti*” but also “*Aedes albopictus*” (better known as the tiger mosquito and also widespread in Italy). Other species of Aedes mosquitoes (*A. africanus*, *luteocephalus*, *furcifer*, and *taylor*) are capable of transmitting the virus as well. The reservoir host is not known, but it is reasonable to hypothesize that it is a monkey [22].

Although primary transmission occurs through vectors, there are—albeit only in a modest percentage of cases—other types of transmission, such as sexual, via transfusion, following transplants, and intrauterine from mother to fetus with congenital infection [22]. Viral RNA has been detected in several bodily fluids, such as plasma, cerebrospinal fluid, and seminal and vaginal fluids one to ten days after infection. The persistence of ZIKV-RNA in sperm has been reported up to 9–10 weeks from the clinical onset of the disease, with the window of sexual transmission still unclear. The presence of ZIKV in vaginal secretions has been identified up to six months after the onset of the disease, a much longer period than in other biological fluids [23].

**Figure 2:**
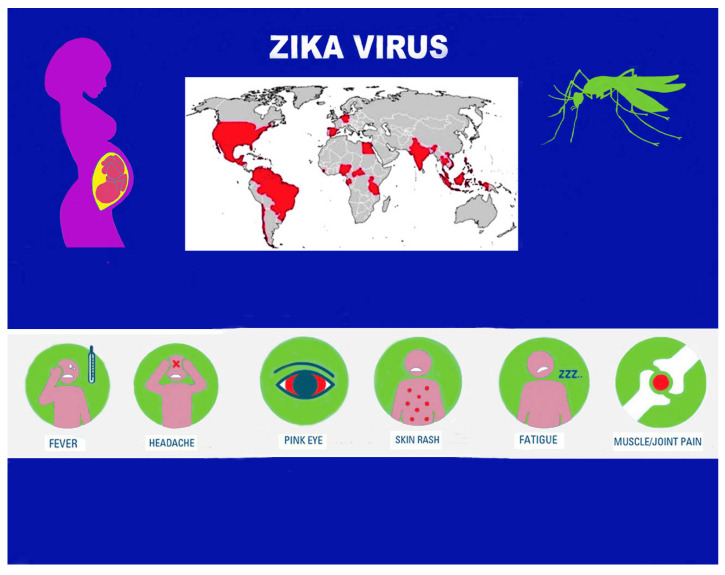
Etiological and epidemiological features, main means of transmission (mosquitos bites and vertical transmission) and clinical symptoms of Zika virus infection.

Vertical transmission was highlighted first in the cases of the Brazilian epidemic affecting women; [24] this was long doubted (there were insufficient previous data on microcephaly) but then definitively accepted after a series of specific studies. Data supporting vertical infection and the causal role of ZIKV in the development of congenital malformations include the finding of ZIKV-RNA or viral antigen in amniotic fluid, the placenta, or the brain tissue of fetuses or newborns with microcephaly diagnosed after death in utero or immediately after birth. Since the virus can cross the placental barrier and cause a congenital (intrauterine) infection, both transmission from mother to fetus and intrapartum transmission are possible [25]. A 2016 study by Brasil P. et al. on infected Brazilian pregnant women showed anomalies such as microcephaly, growth retardation, and fetal death in 29% of fetuses [26]. These findings are confirmed and integrated in a more recent study assessing the prevalence rates of fetal and neonatal disorders in Zika virus-infected pregnancies: central nervous system abnormalities had the highest prevalence, followed by microcephaly, fetal loss, and neonatal RT-PCR positivity for ZIKV [27] (Figure 2).

## 5. Pathogenetic Mechanisms in Pregnancy

Through studies both on pregnant women infected with ZIKV [28] and on pregnant mouse models inoculated with different strains of ZIKV [29], it has been possible to understand what can happen in women who contract ZIKV infection during the first two trimesters of pregnancy (in the third trimester, the infection does not cause significant fetal alterations). A particular tropism of the virus towards the cells of the maternal–fetal interface and, therefore, a transplacental route of infection has been highlighted [28,29].

Placental inflammation (placentitis) represents the fundamental clinical event in the pathogenesis of the vertical transmission of ZIKV infection; placentitis, however, presents non-specific histopathological characteristics that are similar to those described in other placental infections [30]. These features include chronic placentitis, villitis, an increased number of Hofbauer cells, patchy fibrin deposits, increased mononuclear cells in the villous stroma, villous immaturity, edema, hypervascularization, stromal fibrosis, calcifications, and focal necrosis of syncytiotrophoblasts [31].

**Figure 3:**
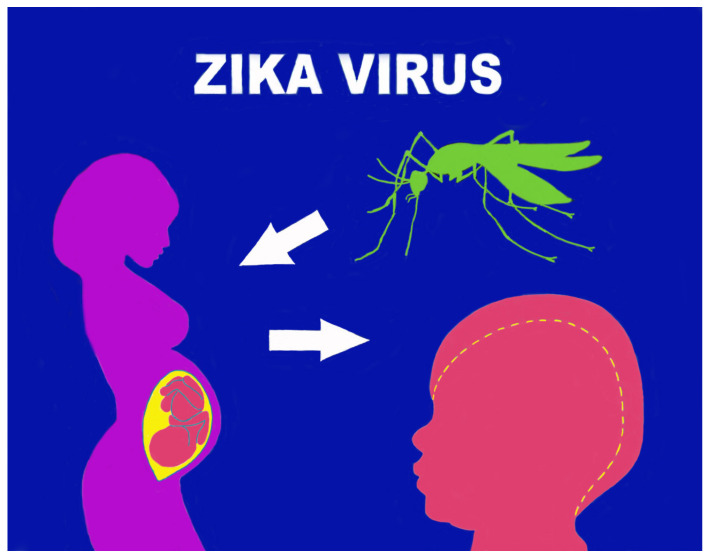
Zika virus infection: maternal–fetal transmission, CZS.

The virus replicates specifically in subgroups of trophoblasts, in fetal endothelial cells, and induces the multiplication of macrophages of the fetal component of the placenta (Hofbauer cells of the villous stroma). Access, via endocytosis, to this type of cell can be amplified by the binding of ZIKV to the tyrosine kinase (RTK) receptor, located on the cell surface and called AXL. Members of the TAM family of RTK receptors (TYRO, AXL, and MER) are believed to be the most likely ZIKV dockers on human cells. Indeed, it has been observed that RTK-TAM receptors not only regulate the homeostasis of mature tissues but can also be found in proliferating progenitor cells in developing neural tissues [32]. The virus thus manages to overcome the fetoplacental barrier, facilitating viral transfer from the placenta to the fetal brain and the specific colonization of the neural progenitors of the fetal cerebral cortex. The overcoming of the fetoplacental barrier has been hypnotized for other pathogens such as human papillomavirus [33,34] and polyomaviruses [35]. This process might be favored by the immunomodulation occurring throughout pregnancy. If ZIKV is contracted in the early stages of pregnancy, it can cause severe placental hypoperfusion and the subsequent impairment of fetal vascularization with neonatal infection and loss of the fetus. However, ZIKV can also cross the placental barrier without causing significant local damage and spread to fetal brain tissue, where it can infect and severely damage neuronal progenitor cells. This situation arises in the case of late-pregnancy infections following the increase in natural immunity induced by IFN λ in the trophoblasts [36]. The infection and death of neural progenitor cells could inhibit neuronal differentiation and explain the cortical thinning, malformation of brain structures, and microcephaly associated with this infection. ZIKV is the only vertically transmitted flavivirus with the potential to infect brain cortical progenitor cells by interfering with cell migration. Vertical transmission of ZIKV can therefore cause serious fetal defects, particularly involving the brain and eye. Recent estimates, based on prospective studies, have calculated a vertical transmission rate of 20–30%, without any correlation to the presence and/or severity of maternal symptoms [31,32,36] (Figure 3).

## 6. Microcephaly and Other Brain Anomalies Due to CZS

### 6.1. Microcephaly

Congenital Zika syndrome is the most severe complication that can occur during pregnancy in women infected with ZIKV; fetuses, regardless of whether the mother is symptomatic or not, have a 5–14% risk of developing CZS and a 4–6% risk of presenting microcephaly [32]. The risk of developing CZS is higher in the first trimester of pregnancy (8–15%), compared to the following two trimesters (4–5%) [32]. The main signs and symptoms associated with the congenital Zika syndrome were microcephaly, parenchymal or cerebellar calcifications, ventriculomegaly, central nervous system hypoplasia or atrophy, arthrogryposis, ocular findings in the posterior and anterior segments, abnormal visual function, and low birthweight for gestational age [37,38,39]. Microcephaly is the first and most notable anomaly found at birth in children born to mothers with ZIKV infection, as it is observed at birth in approximately 80% of children. Microcephaly is defined as a condition where the head circumference of newborns falls below the normal age- and sex-specific standards. Specifically, microcephaly is diagnosed when the head occipitofrontal circumference is below two standard deviations for gestational age and sex, according to the INTERGROWTH-21 standard [36], below the average in relation to sex and gestational age; in the most severe forms, HC can be less than three SDs below the average. Alongside microcephaly, or sometimes even in its absence, brain anomalies may be present, resulting from an interruption of brain development during gestation with consequent skull collapse and disturbance of neuronal and glial migration [32,40]. In a study with seventy-one Brazilian children with prenatal ZIKV infection, the most common abnormalities included calcifications (especially in the cortico-subcortical junction of the white matter), malformations of the cortex, ventriculomegaly, reductions in brain volume, cerebellar hypoplasia, and corpus dysgenesis callosal [41]. Additional brain anomalies that are found, although less frequently, are cerebellar hypoplasia, lissencephaly, and pachygyria (abnormalities in cerebral convolutions) [32]. In addition to microcephaly, other abnormalities associated with congenital ZIKV infection include low birth weight, excessive scalp skin, facial disproportion, swallowing difficulties, hypertonia/spasticity, tremors/convulsions, and hearing impairments. These conditions can result from tissue damage, particularly during the first three months of gestation, resulting in anomalies of macroscopic (malformation disruption) and microscopic (dysplasia) development of the central nervous system, frequently associated with microcephaly [42].

Despite numerous recent studies, the mechanisms of ZIKV infection are still poorly understood. We have already seen how ZIKV infects cells by interacting with cellular receptors and penetrating, via endocytosis, the parasitized cell.

It has been shown that AXL receptors are expressed on proliferating neuroepithelial cells in the brain and retina, they and appear to have increased in neuroepithelial areas in mouse models and human brain organoids, acting as an entry route for ZIKV [43]. These data have highlighted how ZIKV presents tropism for neural progenitor cells (NPCs); once it penetrates the neurogenic brain regions, ZIKV acts by reducing the number of mitotic progenitor cells, inducing cell cycle block and determining, through the activation of caspases, apoptosis. Another mechanism may be to trigger autophagy, presumably in an attempt to evade the host immune system and promote its own replication. ZIKV would therefore appear to act by determining cellular behaviors that alter the proliferation and survival of NPCs during the most critical phases of brain development [44,45,46].

According to studies from India, the main viral culprit responsible for the action of ZIKV in triggering the cellular and molecular mechanisms that determine microcephaly and associated anomalies is the Zika virus envelope protein (E). This protein would affect the normal properties of neural stem cells (NSCs) in children with CZS, causing quiescence in NSCs, and reducing the pool of brain cells, thus leading to microcephaly. The expression of E protein shows maximum quiescence in human fetal neural stem cells (fNSCs), resulting in a marked accumulation of such cells in the G0/G1 phase of the cell cycle compared to other non-structural ZIKV proteins such as NS2A, NS4A, and NS4B. E protein induces immature differentiation by the induction of pro-neuronal genes in proliferating fNSCs and causes apoptosis in differentiating fNSCs three days after cell differentiation [47,48].

**Figure 4:**
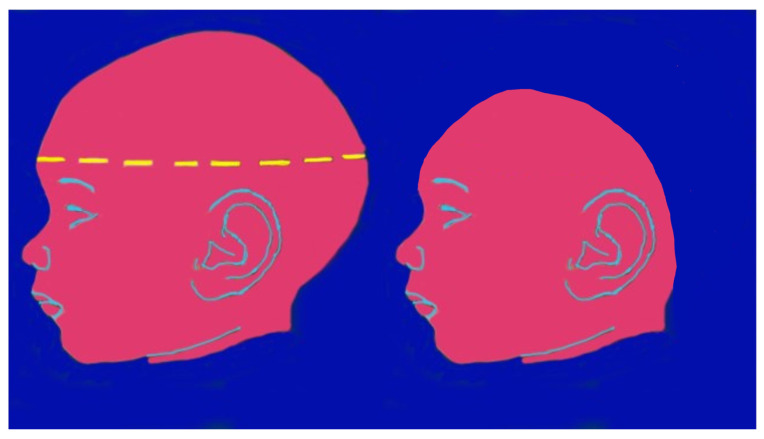
Zika virus infection: microcephaly.

Following a recent study by the CNR Neuroscience Institute and the University of Pisa, a new mechanism has been hypothesized that correlates CZS to damage to the brain development of unborn children. This is based on the intuition that CZS has notable similarities with Rett syndrome (RTT) caused by FOXG1, a protein involved in the development of the cerebral cortex. FOXG1, which encodes the Forkhead box 1 protein, has an important role from the early stages of embryonic development by acting as a transcription factor. This gene transcribes information instructing stem cells to create a precise biological structure and contributes to the development of the telencephalon, an embryonic structure from which the cerebrum is formed. Congenital alterations in gene levels result in “RTT-FOXG1 syndrome”, a clinical situation that recapitulates congenital defects found in “congenital Zika syndrome”, such as microcephaly and other neurodevelopmental conditions [49] (Figure 4).

### 6.2. Orofacial Anomalies

In addition to the typical clinical presentation, there can be manifestations affecting the head and neck region, specifically within the oral cavity. In particular, Zika virus infection can present in this area both as an expression of the so-called congenital Zika syndrome (CZS) [50].

Oral, facial, and dental alterations are common in CZS. Tooth development, known as odontogenesis, begins around the sixth week of intrauterine life and involves cells migrating from the neural crest, which is the same embryonic tissue from which the central nervous system originates. Disturbances during this period can affect the physiology and morphology of dental tissues, leading to changes in their internal and external anatomy [37]. Among children with microcephaly associated with ZIKV, notable changes in the orofacial region include altered eruption chronology, dental morphology, oral structures, and gnathic bones, as well as other modifications such as hypersalivation, child irritation, and anterior open bite [50]. Studies have shown delayed tooth eruption in most evaluated children, with a range of 17.8% to 60.7% experiencing delayed eruption [50,51,52,53,54,55,56,57,58]. The average age of the first tooth eruption ranged from 8 to 12.3 months [52,53,54,59]. In a recent prospective case series including 34 children, the mean chronological age of eruption of the first primary tooth was 12.4 months (SD = 2.9). At the age of 12 and 18 months, 33.3% (*n* = 10) and 13.3% (*n* = 4) of the children had no erupted primary tooth, respectively. Alteration in the sequence of tooth emergence was observed in 41.1% (*n* = 14) of the children [60]. Delayed eruption and changes in the sequence of tooth emergence, affecting the first tooth in particular, may be more pronounced in cases with severe neuro-psychomotor damage [53,61]. However, in many cases, clinically absent teeth are present in the jaws, resulting in a condition known as oligodontia [62]. Regarding dental morphology, opacity is the most observed developmental defect, followed by enamel hypoplasia [51]. Other reported dental changes include microdontia, agenesis, and fusion [53,54,55,56,57,60].

During typical childhood development, the palate tends to be wide and flat [63]. Oral structures and gnathic bones are frequently affected in CZS. According to a recent Brazilian cross-sectional, observational study on 61 patients with microcephaly/CZS, a narrow palate and tongue anterior projection are significantly more prevalent in the microcephaly/CZS group compared to normal development. The microcephaly group also demonstrates reduced measurements of face width, mandible width, height of the faces upper third, and monthly growth of the cephalic perimeter [50]. These alterations in palate shape may result from ZIKV impact on cranial neural crest cells, affecting normal craniofacial development [64]. Additionally, a narrow palate might be associated with the hypotonia of orofacial musculature commonly observed in children with CZS. Tongue posture abnormalities, the presence of narrow palatine vaults, and alterations such as macroglossia and ankyloglossia are frequently reported by many authors [36,39,40,41,42]. Microcephaly caused by ZIKV contributes to orofacial disproportions, decreased cranium size, retrognathia, and micrognathia [55]. Changes in resting lip posture, increased tongue tonus, decreased cheek tonus, and abnormal insertion of the upper labial frenulum have also been documented [56,65]. Children with CZS often experience feeding disorders, swallowing difficulties, and a higher prevalence of low weight [56,58,65]. The shape of the palate has been significantly associated with dysphagia in CZS patients [53,55]. Dysphagia is linked to the loss of voluntary activity during the oral swallowing phase, commanded by the cerebral cortex [50], and is a consequence of oral motor dysfunctions that can lead to severe nutritional complications [56]. Hypersalivation, irritability, and gingival pruritus are also reported symptoms [54,56]. These findings suggest that children with CZS may be prone to developing malocclusions, with a considerable proportion already exhibiting anterior open bite [60]. Additionally, bruxism has been observed in one fifth of patients with microcephaly [61]. Mouth breathing, functional habits, breastfeeding problems, intake of ultra-processed foods, and low weight are more prevalent in children with CZS compared to healthy children [56]. Clinical evaluations of CZS patients with mild and moderate/severe oropharyngeal dysphagia have shown poor lip seal, lack of coordination in sucking–swallowing–breathing, and an absence of pauses to breathe while sucking. Appropriate lip closure is significantly associated with efficient labial sealing and successful swallowing [65].

## 7. Conclusions

Zika virus infection constitutes an infectious disease entity with highly variable epidemiological profiles, both in intensity and geographical spread. In fact, we go from whole years of silence to sudden epidemic outbreaks, mostly local–regional. The geographical spread is very varied, ranging from Africa to the Pacific islands, from Asia to Latin America, with some sporadic episodes in North America and Europe. However, the tropicalization of the climate, the spread of vectors in previously unfrequented areas, and traveling and trading goods at an intercontinental level can lead to a greater geographical expansion of the virus, as it happened for other infectious diseases that had the same characteristics. This, obviously, implies continuous and careful vector surveillance.

The most severe clinical form caused by the Zika virus is linked to infection during pregnancy, which can cause miscarriage or CZS, with the set of symptoms previously described. Therefore, it is essential that pregnant women exposed to the Zika virus undergo screening, as the infection in the mother may be present in an asymptomatic or paucisymptomatic manner and with highly non-specific symptoms. The follow-up of children born to infected mothers is equally important, in order to identify any potential neurological damage as early as possible.
